# SNARER: new molecular descriptors for SNARE proteins classification

**DOI:** 10.1186/s12859-022-04677-z

**Published:** 2022-04-24

**Authors:** Alessia Auriemma Citarella, Luigi Di Biasi, Michele Risi, Genoveffa Tortora

**Affiliations:** Department of Computer Science, University of Salerno, Fisciano, Italy

**Keywords:** SNARE, Protein classification, Machine learning, Random forest, AdaBoost, KNN

## Abstract

**Background:**

SNARE proteins play an important role in different biological functions. This study aims to investigate the contribution of a new class of molecular descriptors (called SNARER) related to the chemical-physical properties of proteins in order to evaluate the performance of binary classifiers for SNARE proteins.

**Results:**

We constructed a SNARE proteins balanced dataset, D128, and an unbalanced one, DUNI, on which we tested and compared the performance of the new descriptors presented here in combination with the feature sets (GAAC, CTDT, CKSAAP and 188D) already present in the literature. The machine learning algorithms used were Random Forest, k-Nearest Neighbors and AdaBoost and oversampling and subsampling techniques were applied to the unbalanced dataset. The addition of the SNARER descriptors increases the precision for all considered ML algorithms. In particular, on the unbalanced DUNI dataset the accuracy increases in parallel with the increase in sensitivity while on the balanced dataset D128 the accuracy increases compared to the counterpart without the addition of SNARER descriptors, with a strong improvement in specificity. Our best result is the combination of our descriptors SNARER with CKSAAP feature on the dataset D128 with 92.3% of accuracy, 90.1% for sensitivity and 95% for specificity with the RF algorithm.

**Conclusions:**

The performed analysis has shown how the introduction of molecular descriptors linked to the chemical-physical and structural characteristics of the proteins can improve the classification performance. Additionally, it was pointed out that performance can change based on using a balanced or unbalanced dataset. The balanced nature of training can significantly improve forecast accuracy.

## Background

SNARE (*Soluble N-ethylmaleimide sensitive factor Attachment protein Receptor*) is a protein superfamily involved in the molecular trafficking between the different cellular compartments [1]. This protein family includes members from yeasts to mammalian cells, evolutionarily conserved. Vesicle-mediated transport is essential for basic cellular processes, such as the secretion of proteins and hormones, the release of neurotransmitters, the phagocytosis of pathogens by the immune system and the transport of molecules from one compartment of the cell to another.Vesicular transport involves membrane receptors responsible for the vescicles recognition, the activation of the membrane fusion and reorganization and the consequent release of the vesicular content in the extracellular space (exocytosis) or inside the cell (endocytosis). Specifically, SNARE complexes mediate membrane fusion during diffusion processes, providing bridging bond between SNARE proteins associated with both membranes [2].

**Fig. 1:**
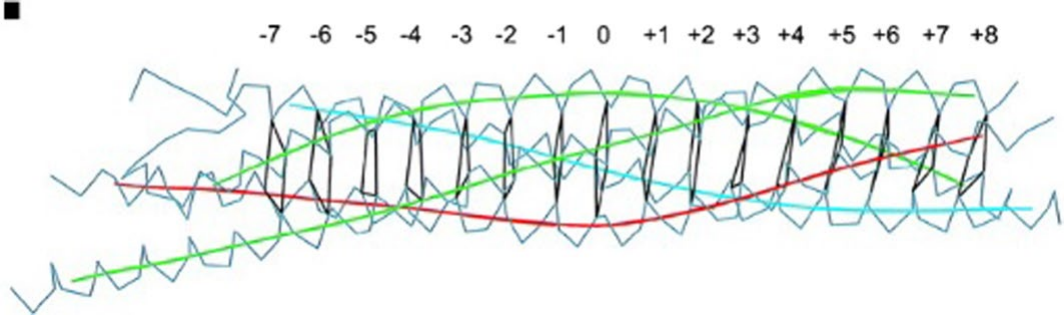
Visualization of the layers of the bundle of the fusion complex between the 4 parallel *α*-helices of the SNARE: 7 upstream layers (layers from − 1 to − 7) and 8 downstream layers (layers from + 1 to + 8) of the ionic layer (the layer 0) [4]

SNARE proteins consist of motifs of 60–70 amino acids containing hydrophobic heptad repeats which form coiled-coil structures. The core of the SNARE complex is represented by 4 *α* helix bundle, as evidenced by the available crystallographic structures [3]. The center of the bundle contains 16 stacked layers which are all hydrophobic, except the central layer “0”, which is called ionic layer and which contains 3 highly conserved glutamines (Q) and a conserved arginine (R) residue (see Fig. 1).

SNARE proteins were initially divided into two categories: vesicle or v-SNARE, which are incorporated into the vesicle membranes, and target or t-SNARE, which are associated with the target membranes. A more recent subdivision is based on their structural characteristics by dividing them into R-SNARE and Q-SNARE. The R-SNARE proteins contain an arginine residue (R) which contributes to the formation of the complex while Q-SNARE proteins contain a glutamine residue (Q) and, according to their position in the bundle of four helices, they are classified in turn as $Q_a$, $Q_b$ or $Q_c$ [4].

In recent years, attention to SNARE proteins has increased due to scientific studies which have shown the implication of SNAREs in some neural disorders for their crucial role in the neuronal and neurosensory release at the level of synaptic endings [5]. The neurotransmitters release is a temporally and spatially regulated process and it occurs thousands of times per minute. In this context, SNARE complexes are continuously subject to tightly regulated assembly and disassembly. Impairment at any stage of this release can lead to hypo or hyperactivity of neurotransmitter release causing dysfunctions which compromise the balance of synaptic communication. There are evidences that these substances seem to be involved in the course of neurodegenerative diseases (such as Alzheimer and Parkinson), in neurodevelopment (autism) and in psychiatric disorders (such as bipolar disorder and schizophrenia as well as depression). Different studies have shown the involvement of mutated or not properly regulated SNARE genes in the development of these disorders [6–11].

Nowadays the protein sequences collection is constantly growing. There is a need to have efficient classification systems able to define the functionality of a protein based on its chemical-physical properties and to label the sequence with greater precision. The more information we can gather about a certain protein, the better our ability to fit it into a more complex biological framework. This is evident and useful especially when considering a protein with an initially unknown function. The most used approach consists in evaluating whether there are functional motifs and domains in the protein which allow to characterize it starting from its amino acid sequence and evaluating its belonging to a protein family in which the members have similar three-dimensional structures, similar functions and significant sequence similarities. Knowledge of the protein family representatives is therefore necessary to define their role and their mechanisms in a specific physiological and pathological biological path. High-throughput sequencing techniques generate lots of big data belonging to different biological domains, including protein sequencing [12]. These huge amounts of data (up to petabytes) must be computationally analyzed with ever newer techniques for the identification of different genomic and protein regions. The current challenge is to contribute to this post-sequencing analysis and classification and to ensure greater precision in the available protein sequences discrimination.

The importance of the evolutionary SNAREs super-family is strictly connected to their role in different cellular functions and different pathological conditions [13, 14], which push researchers to deepen their recognition in the biological pathways.

### Related works

Since SNARE proteins are involved in numerous biological processes, studies have slightly increased in recent decades in order to identify and classify these proteins but the papers dealing with this topic are still few. In the literature there are documents that are based on different techniques, ranging from statistical models to the use of convolutional neural networks.

Kloepper et al. [15] have implemented a web-based interface which allows the new sequences submission to the Hidden Markov Models (HMM) for the four main groups of the SNARE family, in order to classify SNARE proteins based on sequence alignment and reconstruction of the phylogenetic tree. For their study, a set of $\sim$150 SNARE proteins is used in conjunction with the highly conserved motif which is the sequence pattern signature representing the family of SNARE proteins. For SNARE proteins, this motif is an extended segment arranged in heptad repeats, a structural motif consisting of a seven-amino-acid repeating pattern. The extraction of HMM profiles, which allow to identify evolutionary changes in a set of correlated sequences, returns information on the occupancy and position-specific frequency of each amino acid in the alignment. Using this method, the authors are able to obtain a classification accuracy of at least 95% for nineteen of the twenty HMM profiles generated and to perform a cluster analysis based on functional subgroups.

Nguyen et al. [16] have disclosed a model with two-dimensional convolutional network and position-specific scoring matrix profiles for the SNARE proteins identification.The authors used multiple hidden layers for their models, in particular 2D sub-layers such as zero padding, convolutional, max pooling and fully-connected layers with different number of filters. Their model achieves a sensitivity of 76.6%, an accuracy of 89.7% and a specificity of 93.5%.

More recently, in 2020, *Guilin Li* [17] has proposed an hybrid model which combines the random forest algorithm with the oversampling filter and 188D feature extraction method. His work proposes different combinations of feature extraction methods, filtering methods and classification algorithms such as KNN, RF and AdaBoost for the classification of SNARE proteins. Since those results are shown only graphically, it is not possible to have a clear comparison with our results.

## Methods

### Dataset preparation

We have constructed two datasets, respectively named DUNI and D128. Both datasets were used for the evaluation of each classifier’s robustness in unbalanced and balanced training environment, in order to avoid learning bias into classification training. In both datasets, SNARE proteins were downloaded from UNIPROT.^1^ For this purpose, we selected all the proteins with molecular function “SNAP receptor activity”, identified with the unique GENE Ontology [18] alphanumeric code GO: 0005484. The dataset DUNI consists of 276 SNAREs and 806 non-SNAREs. On this unbalanced dataset, we applied the subsampling and ovesampling techniques used in [17]. The balanced dataset D128 is composed of 64 SNARE from UNIPROT and 64 non-SNARE protein sequences downloaded from the PDB database.^2^

In order to create a balanced and non-redundant dataset and improve the dataset quality, all SNARE protein sequences in FASTA format have been processed with the CD-HIT (Cluster Database at High Identity with Tolerance)^3^ program which returns a set of non-redundant representative sequences in output. CD-HIT uses an incremental clustering algorithm. In the first analysis, it sorts the sequences in length descending order and creates the first cluster in which the longest sequence is the representative one. Then the sequences are compared with the clusters representatives. If the similarity with a representative is above a certain threshold, the sequence will be grouped in that cluster. Alternatively, a new cluster is created with that sequence as the representative [19]. The similarity threshold chosen was 25%. This step is very important, since it allows the removal of sequences which exceed the similarity threshold and that could invalidate the analysis causing unwanted bias. Sequence similarity is measured by the similar residues percentage between two sequences. The lower the sequence similarity, the greater the likelihood of having representative proteins in the dataset which consequently show no redundancy [20].

### Feature extraction methods

In order to analyze the data deriving from protein sequences with ML techniques, a numerical representation is required for each amino acid in the protein. For this reason, a series of numerical parameters are often used which act as chemical-physical and structural descriptors of proteins. The combination of a different set of carefully chosen descriptors increases classification efficiency and allows predicting functional protein families [21].

So there are some feature extraction methods commonly used in machine learning. Identifying the right features for machine learning-based protein classification is one of the open issues in this field. The right features combination is important to ensure greater classifier model accuracy [22].

In the literature, over the years, many indices and features of amino acids have been identified for classification methods, such as amino acid composition (AAC), auto-correlation functions [23] or pseudo amino acid composition (PseAAC) [24].

We chose the following four descriptors to compare our SNARER descriptors with those currently used in the SNARE proteins classification.

- GAAC (*Grouped amino acid composition*) groups the 20 amino acids into five groups based on their chemical-physical properties and calculates the frequency for each of the five groups in a protein sequence. Specifically, the five groups are the following: positive charge (K, R, H), negative charge (D, E), aromatic group (F, Y, W), aliphatic group (A, G, I, L, M, V) and uncharge (C, N, P, Q, S, T) [25].
- CTDT (*Composition/Transition/Distribution*) represents the amino acid composition patterns distribution of a specific chemical-physical or structural property in the protein sequence. The final T represents the transition between three types of patterns (neutral group, hydrophobic group and polar group) of which the percentage of occurrence frequency is calculated [25].
- CKSAAP are sequence-based features which, given a sequence, count all adjacent amino acid pairs, considering k-spaced amino acid pairs. Since there are 20 amino acids, for each value of k (from 0 to 5) there are 400 possible pairs of amino acids, for a total of 2400 features [26].
- 188D features constitute a features vector of which the first 20 represent the frequencies of each amino acid while eight types of chemical-physical properties (such as hydrophobicity, polarizability, polarity, surface tension, etc) allow us to calculate the remaining 168 features. In fact, for each type of property 21 features are extracted [27].

For our purpose, we have selected 24 descriptors, 19 of which come from AAindex, i.e., the Amino Acid index database [28]. They are extracted manually, on the basis of the chemical-physical, electrical and energy charge characteristics of the SNARE proteins, according to their principal biological information already known in the literature. We chose features that consider the propensity of individual amino acids to create helixes, sheets and coils. Since there is mainly an helix structure in the SNARE proteins, we opted to evaluate features related to this structure. Others features are related to solvent accessibility, to the ability to interact with the surrounding environment and energy effects of amino acid residues in SNARE proteins.

In this work, we opted to choose these subset of descriptors to assess their behavior in the presence of features that are already widely used in the literature. The other four descriptors (i.e., Steric parameter, polarizability, Volume, Isoelectric point, Helix probability, Sheet probability and Hydrophobicity) are the amino acid parameter sets defined by Fauchere et al. [29]. They are all listed in Table 1.

**Table 1:** The SNARER descriptors

Code	Description	Source
ARGP820102	Signal sequence helical potential%	AAindex [28]
CHAM830101	The Chou-Fasman parameter of the coil conformation	
CHAM830107	A parameter of charge transfer capability	
CHAM830108	A parameter of charge transfer donor capability	
CHOP780204-CHOP780206	Normalized frequency of N-terminal helix-non helical region	
CHOP780205-CHOP780207	Normalized frequency of C-terminal helix-non helical region	
EISD860101	Solvation free energy	
FAUJ880108	Localized electrical effect	
FAUJ880111	Positive charge	
FAUJ880112	Negative charge	
GUYH850101	Partition energy	
JANJ780101	Average accessible surface area	
KRIW790101	Side chain interaction parameter	
ZIMJ680102	Bulkiness	
ONEK900102	Helix formation parameters (delta delta G)	
	Steric parameter	Fauchere et al. [29]
	Polarizability	
	Volume	
	Isoelectric point	
	Helix probability	
	Sheet probability	
	Hydrophobicity	

We used iFeature [25] for feature extraction of GAAC, CKSAAP and CTDT and MSFBinder [30] for 188D.

### Classification algorithms

The work of *Guilin Li* [17] is based on the descriptors GAAC, CTDT, 188D and CKSAAP and subsampling and oversampling methods. This study compared three machine learning algorithms, AdaBoost, K-Nearest Neighbor classifier and Random Forest to predict SNARE proteins. They achieve high accuracy in combination with all four feature extraction methods. In particular, the Random Forest algorithm with oversampling filter and 188D feature extraction approach had the best performance.

Following [17], given the high performances reported, we used the same three classification algorithms to evaluate how accuracy varies with the SNARER descriptors utilization. Thus, we have compared three different ML algorithms: AdaBoost (ADA) K-Nearest Neighbor classifier (KNN) and Random Forest (RF).

- AdaBoost is a machine learning meta-algorithm used in binary classification. AdaBoost is an adaptive algorithm which generates a model that is overall better than the single weak classifiers, adapting to the weak hypothesis accuracy and generating one weighted majority hypothesis in which the weight of each weak hypothesis is a function dependent of its accuracy. At each iteration, a new weak classifier is sequentially added which corrects its predecessor until a final hypothesis with a low relative error is found [31].
- KNN is a supervised learning algorithm used for predictive classification and regression problems. The basis of the operation of this algorithm is to classify an object based on the similarity between the data, generally calculated by means of the Euclidean distance. In this way the space is partitioned into regions according to the learning objects similarity. This algorithm identifies a collection of k objects in the training set that are the most similar to the test object. So, a parameter k, chosen arbitrarily, allows us to identify the number of nearest neighbors, considering the k minimum distances. The prevalence of a certain class in this neighborhood becomes a forecast in order to assign a label to the object [32].
- RF is a supervised learning algorithm that combines many decision trees into one model by aggregation through bagging. The final result of the RF is represented by the class returned by the largest number of decision trees. In particular, the random forest algorithm learns from a random sample of data and trains on random characteristics subsets by splitting the nodes in each tree [33].

### Training and validation sessions

All training sessions were conducted with Weka ML Platform (*Waikato Environment for Knowledge Analysis*), a software environment written in Java which allows the application of machine learning and data mining algorithms [34]. In order to speed-up analysis, an ad-hoc grid, based on the map/reduce paradigm, were used to distribute the work across multiple slaves [35]. Both data sets were used as the input for the training step for AdaBoost, KNN and RF classifiers. There were only two possible output classes: SNARE/ NON SNARE. Then, for each training session, we used the following cross-validation values: the range between 10 to 100 for k-fold and between 20 to 80% for hold out. As a result, the *ratio* of the samples in training and validation set is variable. Moreover, in addition to other parameters configured as in [17], we set k = 1 and Euclidean distance for the distanceFunction of KNN; for the AdaBoost algorithm, default values are weightThreshold = 100 and numIterations = 10, whilst for RF numIterations = 100.

The complete working set was composed of four logical parts: *i)* DUNI non-filtered; *ii)* DUNI oversampled; *iii)* DUNI subsampled; *iv)* D128 non-filtered. For each training session, we generated 10 k-fold variants and 7 hold out variants. Then, for each variant we computed 100 training sessions of each of the three classifiers for each of the four descriptors. Thus, we distributed up to 836.000 training sessions among the distributed computing environment.

### Performance measurement

We evaluated the ML models (Random Forest, AdaBoost and KNN) on the unbalanced dataset DUNI and on the balanced dataset D128. In order to estimate the prediction performance of the three ML algorithms, accuracy (ACC), sensitivity (SN) and specificity (SP) were used. The chosen metrics are described in the equations below:

1 $$\begin{aligned} Accuracy&= \frac{TP+TN}{TN+FP+FN+TP} \end{aligned}$$

2 $$\begin{aligned} Sensitivity&= \frac{TP}{TP+FN} \end{aligned}$$

3 $$\begin{aligned} Specificity&= \frac{TN}{TN+FP} \end{aligned}$$

where TP, TN, FP and FN represent the number of true positives, true negatives, false positives and false negatives, respectively. Sensitivity is the percentage of positive entities correctly identified. Specificity measures the proportion of negative entities that are correctly identified.

In a biological sense, having a TP in our experiment means finding that a protein cataloged as a SNARE is recognized by the classifier as a SNARE.

The feature extraction methods were initially evaluated separately (GAAC, CKSAAP, CTDT and 188D) on the datasets D128 and DUNI, and subsequently these methods were extended with the SNARER descriptors addition disclosed in this work, here identified as extended classes *“ext”*.

## Results and discussion

We used the SNARER descriptors and the three chosen ML algorithms on the unbalanced dataset DUNI and on the balanced dataset D128.

We have first considered four feature sets (GAAC, CTDT, CSKAAP and 188D) separately and then each one in combination with the SNARER descriptors class, identified with *“.ext”*. The classification performances were evaluated with three metrics: average accuracy (ACC), average sensibility (SN) and average specificity (SP).

### Results on the unbalanced dataset DUNI

**Fig. 2:**
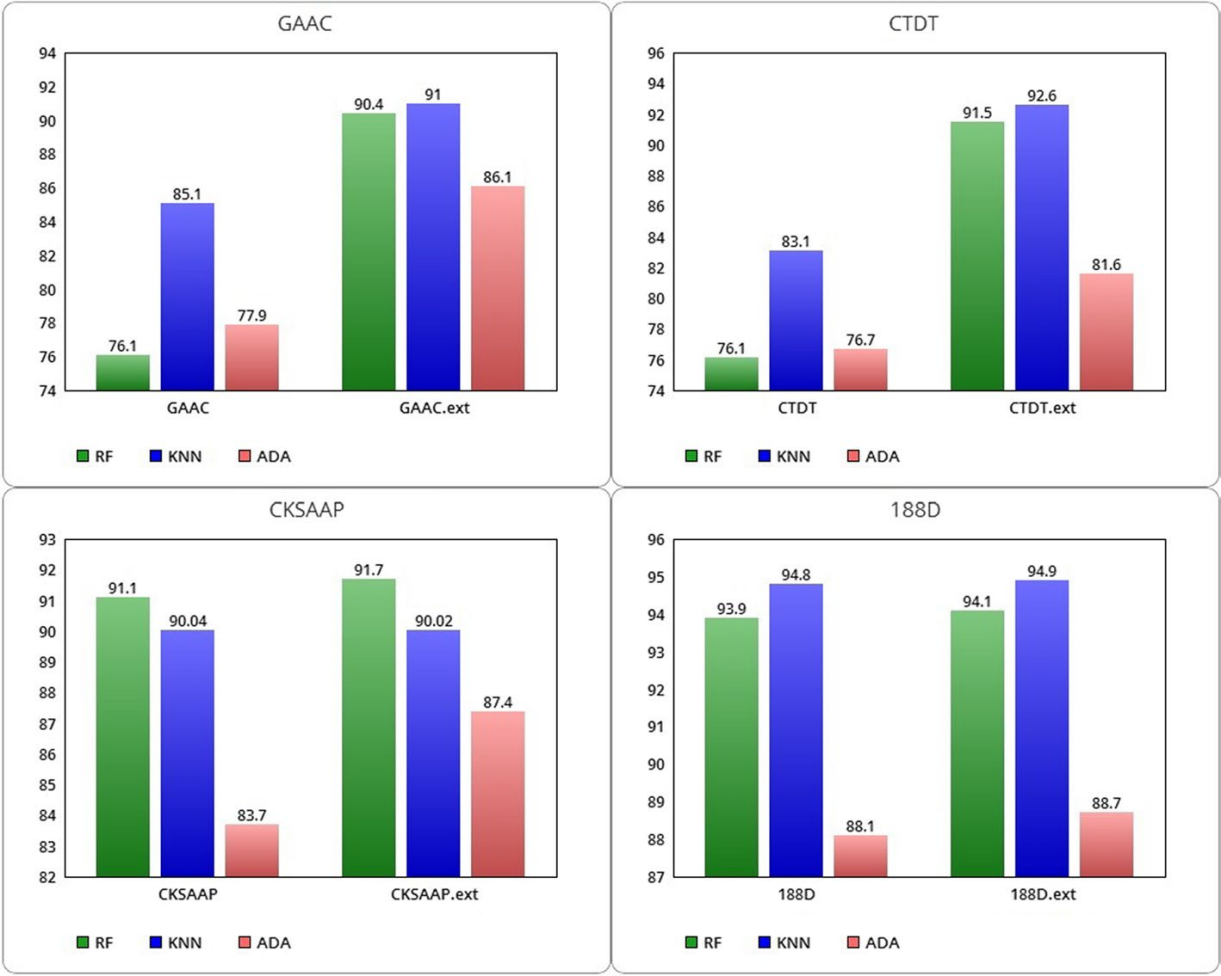
Comparison between GAAC, CTDT, CKSAAP and 188 D ACC with related extended classes with SNARER (on DUNI dataset)

Below, we have reported the experimental results conducted on the DUNI dataset. Related to the four protein feature extraction methods GAAC, CTDT, CKSAAP and 188D, the average ACCs for the ML algorithms are included in a range between 76 and 94.9%. In Fig. 2, histograms are used for the graphical comparison of the three ML techniques.

As shown in Table 2, the introduction of the SNARER class brings a strong improvement in combination with all the considered protein feature extraction methods. Overall, the best average accuracy is achieved with the KNN model and with the 188D feature set and the SNARER class combination. This combined model achieves also the best average SP while the best average SN is obtained with the RF model trained using both GAAC and CTDT features separately (see Table 3).

**Table 2:** Performance of average ACC on the DUNI dataset

	Accuracy		
	RF	KNN (%)	ADA (%)
GAAC	76.1	85.1	77.9
GAAC.ext	**90.4**	**91**	**86.1**
CTDT	76.1	83.1	76.7
CTDT.ext	**91.5**	**92.6**	**81.6**
CKSAAP	91.1	90.04	83.7
CKSAAP.ext	**91.7**	90.02	**87.4**
188D	93.9	94.8	88.1
188D.ext	**94.1**	**94.9**	**88.7**

The highest values are shown in bold

**Table 3:** Performance for average SN and SP on the DUNI dataset

	Sensitivity			Specificity		
	RF	KNN (%)	ADA (%)	RF (%)	KNN (%)	ADA (%)
GAAC	**99.8**	90.3	83.6	7	7	**61**
GAAC.ext	97.2	**94.5**	**94.8**	**70.7**	**80.6**	60.6
CTDT	**99.8**	89.1	83.3	7.1	65.6	**57.6**
CTDT.ext	96.6	**94.6**	**91.1**	**76.4**	**87**	54
CKSAAP	97.8	98	89.9	71.7	66.7	65.5
CKSAAP.ext	97.8	98	**92**	**74**	66.7	**74**
188D	**97**	**96.6**	92	85	89.5	76.7
188D.ext	96.8	96.5	**92.4**	**86.3**	**90.1**	**78**

The highest values are shown in bold

For RF, SN decreases imperceptibly in the extended classes with the new descriptors, remaining unchanged for the CKSAAP method. In contrast, for RF, SP increases with the extended classes, notably especially for the GAAC and CTDT extraction methods.

The SN of KNN increases significantly in the extended classes referred to GAAC and CTDT and remains substantially unchanged for CKSAAP and 188D. The same trend is also shown for the SP of KNN, with a slight improvement of the extended 188D class. For the AdaBoost algorithm, we observe an increase in SN, mostly for the extended GAAC and CTDT classes, which however show a decrease in SP. The SP ADA, instead, increases for the extended classes CKSAAP and 188D. Overall, on the unbalanced dataset the use of extended classes with our SNARER descriptors results in an improvement in accuracy for the GAAC, CTDT, CKSAAP and 188D classes of all three ML models, except for KNN trained with CKSAAP. All selected ML algorithms achieve SN greater than 83%, with the best SN of 99.8% RF achieved by GAAC and CTDT without extension.

By introducing the SNARER class for all four feature sets, the SN settles in a range between 91.1% of the ADA algorithm with the CTDT class and 98% of the KNN algorithm with the extended CKSAAP class. Regarding the SP, without the SNARER’s descriptor extension, the range extends from a minimum of 7% of RF and KNN algorithms for the GAAC class to a maximum of 89.5% of KNN trained with the 188D feature set. With the SNARER class addition, an SN of 54% of ADA with CTDT feature set is obtained at a maximum of 90.1% of KNN trained on the dataset with 188D feature set. More specifically, the KNN model using the 188D extended class with SNARER descriptors, achieves better performance in all metrics except for SN, where the RF model trained with the GAAC features obtains the highest value.

In conclusion, on the unbalanced DUNI dataset, the new SNARER descriptors class guarantees an improvement in terms of ACC in combination with all four tested features sets and a clear improvement of SN and SP of some ML tested algorithms.

#### Results on the unbalanced dataset DUNI with oversampling and with subsampling

Because the dataset DUNI is unbalanced, we have adopted subsampling and oversampling techniques.

With the oversampling technique on the DUNI dataset, the SNARER class produces a strong improvement in accuracy, more for the extended GAAC and CTDT classes for the three ML models RF, KNN and ADA, while the contribution to the CKSAPP and 188D feature sets remains substantially unchanged (as shown in Table 4). The same behavior is common to the average SN and average SP calculated for RF, KNN and ADA (see Table 5). Applying the subsampling technique to the DUNI dataset, we observe the same trend for SN but with a slight decrease, around 2% -4%, of the values when considering the extended classes CKSAAP and 188D. The same decrease value is also present for the average SPs of the same classes (see Table 6).

**Table 4:** Performance of the average ACC on the DUNI dataset with oversampling and subsampling

	Oversampling			Subsampling		
	RF	KNN (%)	ADA (%)	RF (%)	KNN (%)	ADA (%)
GAAC	94.7	96.3	73.12	75.2	79.2	72.6
GAAC.ext	**98.03**	**98.44**	**85.02**	**91.8**	**86.4**	**82.6**
CTDT	93.9	96.1	70.4	74.6	78.1	71.7
CTDT.ext	**98**	**98**	**86.3**	**90.6**	**89.7**	**86.4**
CKSAAP	**99.07**	**98.67**	84	**93.1**	**84.4**	83.5
CKSAAP.ext	99.01	98.6	**89.1**	79	84.2	**87.3**
188D	98.5	98.90	89.5	93.1	**95**	86.6
188D.ext	98.5	**98.95**	**89.6**	**93.5**	94	**89.3**

The highest values are shown in bold

**Table 5:** Performance for average SN and SP on the DUNI dataset with oversampling

	Sensitivity			Specificity		
	RF (%)	KNN (%)	ADA (%)	RF (%)	KNN (%)	ADA (%)
GAAC	91.9	95	74.9	97.5	97.6	71.3
GAAC.ext	**96.6**	**97.8**	**88.4**	**99.4**	**99.1**	**81.6**
CTDT	88.9	94	68.8	98.8	98.2	72
CTDT.ext	**96.4**	**96.9**	**78.4**	**99.5**	**99.2**	**94.3**
CKSAAP	**99**	**99.2**	80.8	99.2	98.2	87.2
CKSAAP.ext	98.7	99.1	**86.2**	**99.3**	98.2	**92**
188D	97.5	98.3	**90.2**	**99.7**	99.5	88.8
188D.ext	**97.7**	98.3	89.4	99.3	**99.7**	**89.9**

The highest values are shown in bold

**Table 6:** Performance for average SN and SP on the DUNI dataset with subsampling

	Sensitivity			Specificity		
	RF (%)	KNN (%)	ADA (%)	RF (%)	KNN (%)	ADA (%)
GAAC	75.7	76.1	73.6	74.6	82.2	71.7
GAAC.ext	**88.8**	**85.5**	**80.8**	**94.9**	**87.3**	**84.4**
CTDT	78.3	76.4	73.9	71	79.7	69.6
CTDT.ext	**86.6**	**88.4**	**81.9**	**94.6**	**90.9**	**90.9**
CKSAAP	**90.9**	**98.6**	83.3	**95.3**	70.3	83.7
CKSAAP.ext	76.4	98.2	**83.7**	81.5	70.3	**90.9**
188D	90.9	**95.3**	88	**95.3**	94.6	85.1
188D.ext	**92**	93.1	**88.8**	94.9	**94.9**	**89.9**

The highest values are shown in bold

### Results on the balanced dataset D128

**Fig. 3:**
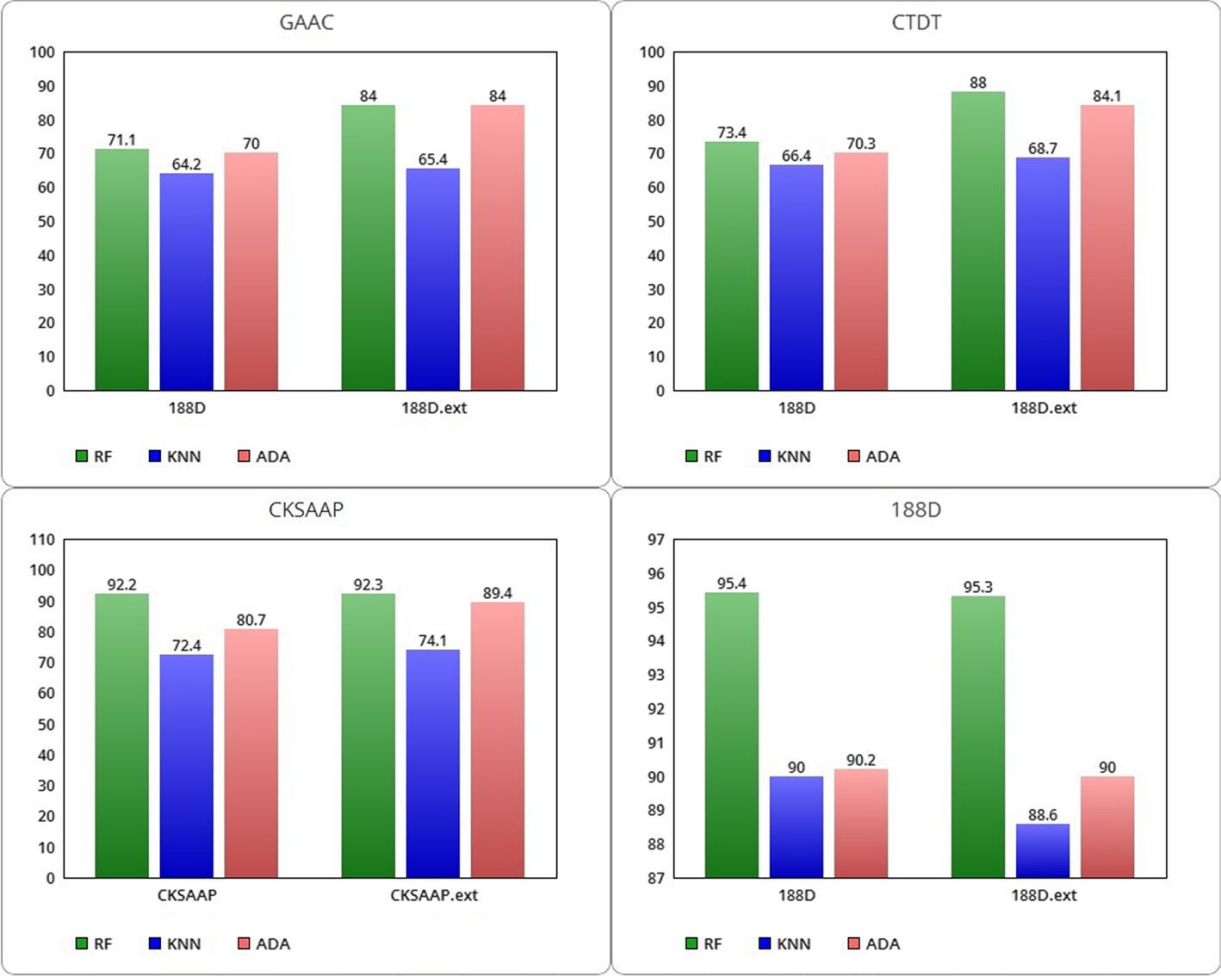
Comparison between GAAC, CTDT, CKSAAP and 188D ACC with related extended classes with SNARE (on D128 dataset)

Below we present the obtained classification results on the balanced dataset D128, with and without the addition of the SNARER descriptors. Table 7 reports the average accuracy performances of the ML algorithms without considering the SNARER descriptors in the balanced D128 dataset. In addition, histograms are depicted graphically in Fig. 3: RF varies from a minimum of 71.1% with the use of the GAAC class to a maximum of 95.4% with the 188D class; KNN settles between a minimum of 64.2% with the use of GAAC to a maximum of 90% with the 188D class; ADA varies from a minimum of 70% with GAAC to a maximum of 90.2% trained on the 188D class. Extended classes with SNARER descriptors shift these average ACC rates. In particular, RF varies from a minimum of 84% using the extension with GAAC to a maximum of 95.3% with the 188D class. KNN starts from a minimum of 65.4% with the extended GAAC class and reaches a maximum of 88.6% with the extended 188D class. ADA varies in a range between 84% with the GAAC.ext class to a maximum of 90% with the combined class 188D.

**Table 7:** Performance of average ACC for the D128 dataset

	Accuracy		
	RF (%)	KNN (%)	ADA (%)
GAAC	71.1	64.2	70
GAAC.ext	**84**	**65.4**	**84**
CTDT	73.4	66.4	70.3
CTDT.ext	**88**	**68.7**	**84.1**
CKSAAP	92.2	72.4	80.7
CKSAAP.ext	**92.3**	**74.1**	**89.4**
188D	**95.4**	**90**	**90.2**
188D.ext	95.3	88.6	90

The highest values are shown in bold

By comparing the evaluated average ACCs, the SNARER class addition improves the classification performance in relation to the GAAC, CKSAAP and CTDT feature extraction methods while there is a slight decrease in the average ACCs for the 188D feature extraction class. Further analysis should be conducted to understand the reason for this decrease. In particular, the best classification results are obtained with the RF algorithm.

With the extended feature extraction methods, we can note that for the RF algorithm SN increases with GAAC and CTDT while it remains fundamentally unchanged for the other two descriptor classes (see Table 8). Also SP increases showing the same behavior. For the KNN algorithm, SN decreases for the GAAC and CTDT classes by 3% and 1% for the 188D class while it increases by 2% for the CKSAAP class. The SP of KNN instead increases for all classes except 188D, with a decrease of about 2%. ADA improves in terms of SN on all extended classes while it decreases in SP by 0.8% when applied on the extended class 188D.

**Table 8:** Performance for average SN and SP on the D128 dataset

	Sensitivity			Specificity		
	RF (%)	KNN (%)	ADA (%)	RF (%)	KNN (%)	ADA (%)
GAAC	80.1	**65.7**	74.5	62.2	63	65.4
GAAC.ext	**84**	62.2	**88.6**	**83.9**	**69**	**79.2**
CTDT	74.7	**70.4**	70	72.2	62.3	70.5
CTDT.ext	**87.6**	64.7	**84.7**	**88.3**	**73**	**83.4**
CKSAAP	89.7	55.4	80.2	95	89.4	81.3
CKSAAP.ext	**90.1**	**57**	**89.5**	95	**91.2**	**89.4**
188D	**95.7**	**89**	88.5	95.1	**91**	**92**
188D.ext	95.5	88	**88.8**	95.1	89.2	91.2

The highest values are shown in bold

### Comparison between the DUNI and the D128 datasets

Carrying out experiments on unbalanced datasets or balanced datasets affects the automatic learning of the different ML algorithms. In fact, it has been observed that when tests are performed on an unbalanced dataset, greater accuracy is achieved since the classification of each test sample towards the majority class prevails [36]. Consequently, choosing a balanced dataset for training tests can lead to a higher quality of classification predictions. In the case of binary classifications, the coefficient of correlation between the true class and the expected class can be calculated, dealing with them as two binary variables. Since the ACC calculation is sensitive to the imbalance class in order to compare the DUNI and D128 datasets, following the SNARER descriptors introduction, we have used the Matthews Correlation Coefficient (MCC) [37]. In this context, we started from the hypothesis that the proportion of correct predictions (accuracy) are not useful when the two classes have different sizes. In this case the use of MCC is useful. It represents a quality measure also in cases where the datasets have different sizes. MCC varies in the range $[-1; 1]$. When the MCC value is 1, it indicates a perfect forecast. If it returns a value of -1 it represents a perfect negative correlation while 0 means that the classifier returns only a forecast no better than a random one. So, MCC considers all four values in the confusion matrix (TP, TN, FP and FN) and a high value (around 1) indicates that both classes are adequately covered, even if one is disproportionately under (or over) represented.

4 $$\begin{aligned} MCC = \frac{TP \times TN - FP \times FN}{{\sqrt{{(TP+FP)}{(TP+FN)}{(TN+FP)}{(TN+FN)}}}} \end{aligned}$$

In Table 9, we presented the comparison between the MCC metrics for RF, KNN and ADA trained on the DUNI and D128 datasets with the extended descriptors classes.

**Table 9:** Comparison of MCC for the DUNI and D128 datasets

	Matthews correlation coefficient			
	Dataset	MCC RF	MCC KNN	MCC ADA
GAAC.ext	DUNI	**0.74**	**0.76**	0.61
	D128	0.69	0.32	**0.70**
CTDT.ext	DUNI	0.77	**0.81**	0.49
	D128	0.77	0.39	**0.70**
CKSAAP.ext	DUNI	0.77	**0.73**	0.69
	D128	**0.86**	0.53	**0.80**
188D.ext	DUNI	0.84	**0.87**	0.70
	D128	**0.91**	0.81	**0.81**

The highest values are shown in bold

**Fig. 4:**
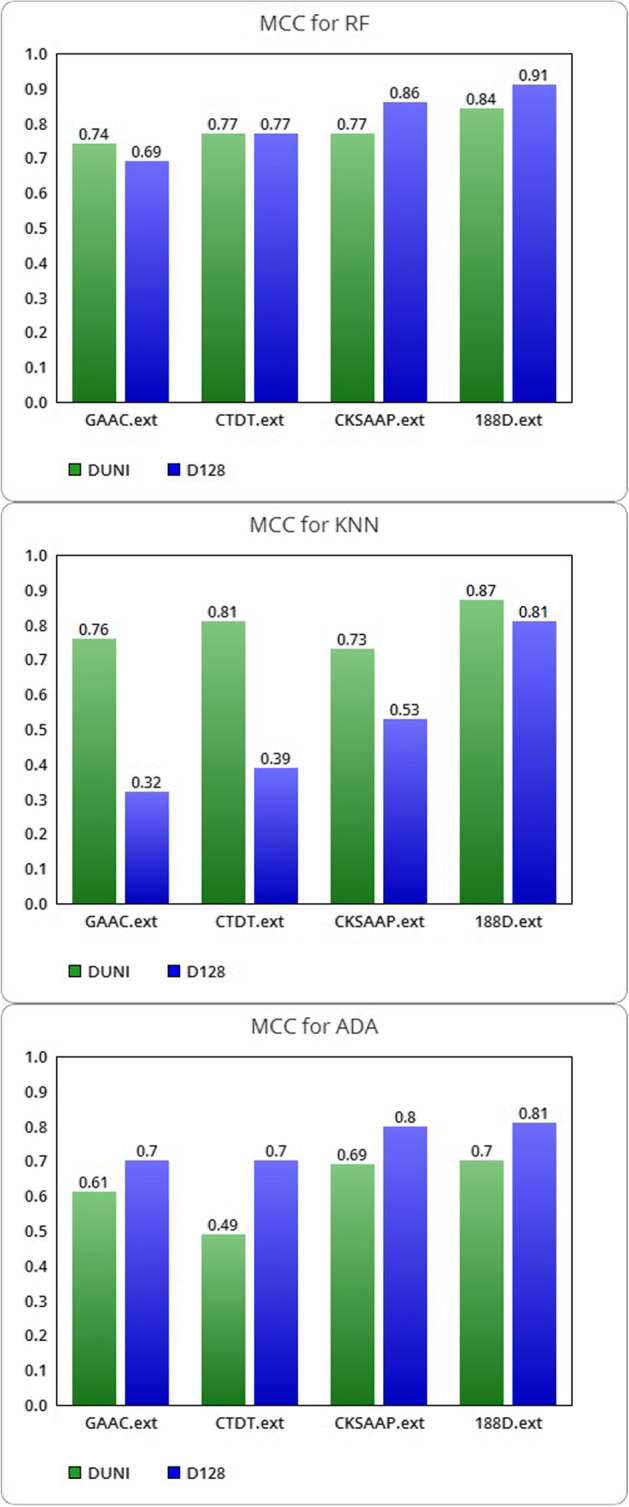
Graphic visualization of MCC for RF, KNN and ADA algorithms

The MCC (see Fig. 4) of RF improves on the balanced dataset, except for a decrease with the GAAC discriminant features and for no change on the CTDT class. The MCC of KNN is lowered for all combined descriptors, significantly for GAAC, CTDT and CKSAAP. In contrast, ADA’s MCC is significantly improved in all four conditions. As a result, we can see how the values of MCC reflect the quality of the classifier input data. Only if the classifier successfully predicted the majority of positive data instances and the majority of negative data instances, MCC can generate a high score. In the presence of DUNI, which is a negatively imbalanced dataset, we have high values in terms of ACC, SN and SP compared to the balanced dataset (see Tables 2, 3, 7, 8). Since it ignores the proportion of positive and negative items, accuracy can produce misleading values for unbalanced datasets [38]. In Table 9, we showed how many MCC values are greater when we evaluate the algorithms on a balanced dataset with no positive and negative samples imbalance. In some circumstances, MCC values remain constant, owing to the classifier’s ability to produce accurate predictions regardless of the *ratio* between classes. The MCC is lower in the case of the KNN algorithm, which reflects the worst performance measured by other measures.

*Area under the receiver operating characteristic* (AUC) and *Area under the precision-recall curve* (AUPRC) were used to assess the performance of the various folds of the conducted experiments. AUC is a metric for evaluating the quality of a classification algorithm that is used in various applications. As a summary measure of the *Receiver operating characteristic (ROC) curve*, the AUC is widely utilized. It is a value between 0 and 1, which considers the area under the curve of the plot of SN versus 1-SP across thresholds. It represents the probability that the model will rate a random positive case higher than a random negative example. AUPRC is the area under the curve of the plot of precision versus SN across thresholds. For imbalanced data, this area is more informative than the AUC and it is thought to be a good measure in order to evaluate the performance of a classifier. AUPRC varies from 0 to 1. In general, a classifier with a high AUC and AUPRC values performs better the given classification task. In Tables 10 and 11, we reported the average values of *Area under the receiver operating characteristic* (AUC) and *Area under the precision-recall curve* (AUPRC) for DUNI and D128 datasets, respectively. On the DUNI and D128 datasets, we can observe that the AUC and AUPRC values for the extended classes are higher. In particular, it is more evident for the balanced dataset D128, pointing out the importance of class balance. Furthermore, these results reflect what was previously seen, regarding the failure to improve the 188D extended class.*Area under the receiver operating characteristic* (AUC) and *Area under the precision-recall curve* (AUPRC) were used to assess the performance of the various folds of the conducted experiments. AUC is a metric for evaluating the quality of a classification algorithm that is used in various applications. As a summary measure of the *Receiver operating characteristic (ROC) curve*, the AUC is widely utilized. It is a value between 0 and 1, which considers the area under the curve of the plot of SN versus 1-SP across thresholds. It represents the probability that the model will rate a random positive case higher than a random negative example. AUPRC is the area under the curve of the plot of precision versus SN across thresholds. For imbalanced data, this area is more informative than the AUC and it is thought to be a good measure in order to evaluate the performance of a classifier. AUPRC varies from 0 to 1. In general, a classifier with a high AUC and AUPRC values performs better the given classification task. In Tables 10 and 11, we reported the average values of *Area under the receiver operating characteristic* (AUC) and *Area under the precision-recall curve* (AUPRC) for DUNI and D128 datasets, respectively. On the DUNI and D128 datasets, we can observe that the AUC and AUPRC values for the extended classes are higher. In particular, it is more evident for the balanced dataset D128, pointing out the importance of class balance. Furthermore, these results reflect what was previously seen, regarding the failure to improve the 188D extended class.

**Table 10:** Average AUC and AUPRC on the DUNI dataset

	AUC			AUPRC		
	RF	KNN	ADA	RF	KNN	ADA
GAAC	0.84	0.81	0.78	0.93	0.89	0.89
GAAC.ext	**0.97**	**0.88**	**0.93**	**0.99**	**0.93**	**0.97**
CTDT	0.84	0.79	0.79	0.94	0.88	0.89
CTDT.ext	**0.97**	**0.91**	**0.90**	**0.99**	**0.95**	**0.96**
CKSAAP	0.98	0.84	0.89	0.99	0.90	0.96
CKSAAP.ext	0.98	0.84	**0.93**	0.99	0.90	**0.97**
188D	0.98	0.94	0.94	0.99	0.96	0.98
188D.ext	0.98	0.94	**0.95**	0.99	0.96	0.98

The highest values are shown in bold

**Table 11:** Average AUC and AUPRC on the D128 dataset

	AUC			AUPRC		
	RF	KNN	ADA	RF	KNN	ADA
GAAC	0.76	0.64	0.75	0.76	0.61	0.74
GAAC.ext	**0.91**	**0.66**	**0.92**	**0.92**	**0.62**	**0.92**
CTDT	0.82	0.66	0.77	0.84	0.62	0.78
CTDT.ext	**0.94**	**0.69**	**0.93**	**0.95**	**0.65**	**0.94**
CKSAAP	0.97	0.72	0.90	0.98	0.70	0.91
CKSAAP.ext	0.97	**0.74**	**0.96**	0.98	**0.72**	**0.96**
188D	0.99	**0.90**	0.97	0.99	**0.87**	0.97
188D.ext	0.99	0.89	0.97	0.99	0.85	0.97

The highest values are shown in bold

### Comparison with the state of the art

In Table 12, we presented the comparison between the proposed method and the literature. The method by [15] is based on Hidden Markov Models (HMM), sequence alignment and phylogenetic tree reconstruction in order to classify SNARE proteins. Nguyen et al. [16] used a model with 2D-CNN and position-specific scoring matrix profiles, while the study of Guilin Li [17] has suggested a hybrid model that incorporates the random forest algorithm, the oversampling filter and the 188D feature extraction approach. As we can see in Table 7, by comparing the use of all extended classes with non extended descriptors, our best result is the combination of SNARER descriptors with CKSAAP feature on the dataset D128 with 92.3% of accuracy, 90.1% for sensitivity and 95% for specificity with the RF. On the other hand, when we considered the results achieved on the balanced D128 dataset with the use of our SNARER descriptors, our highest performance is achieved by the RF algorithm in combination with the 188D features.

**Table 12:** Comparison with reference literature

Authors	Methods	ACC	SP	SN
Kloepper et al.	HMM	95%	–	–
Nguyen et al.	2D-CNN	89.7%	93.5%	76.6%
Guilin Li	188D-RF-oversample	90–95%	95–100%	75–80%
*Our methods*			**Dataset D128**	
(highest value)	RF-188D.ext	95.3%	95.1%	95.5%
(best value)	RF-CKSAAP.ext	92.3%	95%	90.1%

The highest values are shown in bold

188D features include the 20 characteristics about frequencies of each amino acid and 168 features based on using eight types of chemical-physical properties. These features probably strengthen the biological properties of the proteins, allowing to reach high levels of the tested classification algorithms. Further studies are needed to understand the intrinsic reasons for the improvement or decay of some parameters when using 188D features.

## Conclusion

In recent decades, following the exponential increase in data from gene sequencing, it has become necessary to explore different ML techniques for the protein identification, in support of traditional methods [39]. Recent studies on SNARE proteins have shown that their complexes are spoken in the release of neurotransmitters and that their dysfunction is the basis of neurodegenerative, neural developmental and neuropsychiatric disorders. The importance of recognizing them with increasingly precision has a significant biological impact for identifying the aforementioned pathological conditions [40]. The aim of classifying these proteins allows researchers to understand the biological pathways in which they are involved and by increasing their knowledge, they can improve the possible therapeutic approach.

In this work, we tested different feature extraction methods on a balanced and an unbalanced dataset, with and without the new contribution of SNARER descriptors addition, in order to examine the role of balanced and unbalanced training in the classification of SNARE binary proteins. Consequently, we compared the behavior of three ML algorithms (RF, KNN and ADA) on the homogeneous and non-homogeneous datasets.

The ML models were evaluated calculating the ACC, SN and SP average values. Our results showed that the performance of the ML algorithms, with the extension of the SNARER descriptors to the feature sets used, improved on both datasets in terms of average ACC. This improvement is greater for RF, KNN and ADA algorithms with the combination of SNARER descriptors to the 188D class. In particular, our best results on the balanced and non-redundant dataset D128 are 92.3% of ACC, 90.1% for SN and 95% for SP with the RF algorithm and with the extended class *CKSAAP.ext*. By evaluating the MCC for RF, KNN and ADA on both datasets trained with extended feature sets, the ADA algorithm benefited from better performance applied on the balanced dataset. On the contrary, KNN has worsened in terms of performance, reaching a higher value only for the 188D class. Specifically, the algorithms trained on the balanced dataset produce a better MCC, especially for RF and more for ADA, which recovers both in terms of ACC, SP and SN in all the considered tests. KNN, on the contrary, appears to have lower performance in terms of MCC compared to the other algorithms considered.

As future work, it is possible to extend the analysis to also identify the SNARE proteins sub-categories based on their structural features, Q-SNAREs and R-SNAREs. Furthermore, it would be useful to explore the use of different classes of descriptors, also combined with each other, which can guarantee a better classification of the proteins under examination.

## Data Availability

The dataset generated and analysed during the current study are available in the SNARER repository, https://github.com/luigidibiasi/snarer. Access to the repository is public.

## Abbreviations

AAC: Amino acid composition
ACC: Accuracy
ADA: Adaptive Boosting
AUC: Area under the receiver operating characteristic
AUPRC: Area under the precision-recall curve
CTDT: Composition/Transition/Distribution
FN: False negative
FP: False positive
GAAC: Grouped amino acid composition
KNN: K-nearest neighbors
MCC: Matthews correlation coefficient
ML: Machine Learning
RF: Random Forest
ROC: Receiver operating characteristic
SN: Sensitivity SNARE (*Soluble N-ethylmaleimide sensitive factor attachment protein receptor*)
SP: Specificity
TN: True negative
TP: True positive

## Footnotes

1 https://www.uniprot.org/.

2 https://www.rcsb.org/.

3 http://weizhongli-lab.org/cd-hit/.
